# Prevalence and associated determinants of disability among women aged 15–49 years in Somalia: evidence from a 2018/2019 nationwide survey

**DOI:** 10.3389/fpubh.2026.1846271

**Published:** 2026-06-01

**Authors:** Hassan Abdi Ahmed, Mohamed Farah Hassan

**Affiliations:** Center for Graduate Studies, Department of Statistics and Data Analytics, Jamhuriya University of Science & Technology, Mogadishu, Somalia

**Keywords:** individual- and community-level determinants, prevalence, SHDS, Somalia, women aged 15–49

## Abstract

**Background:**

Disability among women of reproductive age is a critical public health issue with significant social and economic implications. Understanding individual- and community-level determinants is essential for targeted interventions.

**Objective:**

This study examines the prevalence and determinants associated with disability among women aged 15–49 years in Somalia.

**Method:**

Data from the Somalia Health and Demographic Survey (SHDS) were analyzed using STATA 17.0, employing a multivariable multilevel logistic regression model to assess individual- and community-level determinants of disability. Multicollinearity was assessed and found not to be a concern. The SHDS used a stratified two-stage cluster sampling design in which enumeration areas were selected with probability proportional to size, followed by systematic selection within each cluster, and inclusion of all eligible individuals, ensuring nationally representative data across population groups. Only statistically significant predictors were reported.

**Findings:**

Overall, 3.05% of women reported a disability. At the individual level, the odds of disability increased with older age (25–34 years: AOR = 1.42; 35–49 years: AOR = 2.17) and among women who were divorced (AOR = 2.34), abandoned (AOR = 4.99), widowed (AOR = 2.20), or never married (AOR = 3.36), while higher education (AOR = 0.51), larger household size (6–10 members: AOR = 0.71; ≥11 members: AOR = 0.67), higher wealth (highest quintile: AOR = 0.63), and health insurance coverage (AOR = 0.04) were protective. At the community level, rural residence (AOR = 0.45) and living in the southern areas (AOR = 0.75) were associated with lower odds of disability compared with urban and northern areas.

**Conclusion:**

The findings underscore the need for targeted, evidence-informed policies that address the determinants associated with disability among women of reproductive age in Somalia. Priority should be given to expanding access to education for women, improving health insurance coverage for disadvantaged and low-income groups, and reinforcing social protection systems for the marginalized groups. In addition, efforts should focus on improving the availability, accessibility, and quality of disability-related health services, with particular emphasis on urban areas and the northern areas, where the likelihood of disability was relatively higher compared with rural residence and the southern areas.

## Introduction

### Study background

Disability is a significant global issue with biological, social, and economic implications, and its rising prevalence worldwide has made it an increasingly important area of study (1–6). Disability is defined as long-term physical, mental, intellectual, or sensory impairments that, when combined with social, environmental, or attitudinal barriers, can limit a person’s full and equal participation in society (7). Globally, the World Health Organization reported that in 2019, at least 2.2 billion people were affected by visual impairment and 1.5 billion by hearing impairment (8). With global population growth, nearly one in four people is projected to experience hearing loss, and approximately 237 million are expected to have moderate to severe visual impairment by 2050 (6, 9).

According to the World Report on Disability, approximately 15% of the global population lives with disabilities, with 80% residing in low- and middle-income countries. The prevalence of disability is also increasing due to factors such as population aging, chronic diseases, changing dietary patterns, substance use, natural disasters, and armed conflict (10). Among different forms of disability, mobility limitations affect around 7% of the population and serve as an important indicator of public health and individual well-being, as they often reduce daily functioning, diminish quality of life, and increase healthcare expenditures (11). Despite its widespread prevalence globally, disability is difficult to measure accurately due to differences in data collection and assessment methods, leading to limited comparable international data and frequent underestimation (11). Globally, mobility disability is more prevalent among women than men, particularly in low- and middle-income countries, and tends to occur at earlier ages among women, suggesting the influence of gender-related risk factors (11, 12). However, disability is a complex concept encompassing conditions that affect physical, mental, and social functioning. Effective policies and interventions require clear definitions and standardized measurement approaches (13).

Evidence from low- and middle-income countries (LMICs) in Asia and East Africa shows substantial variation in the prevalence of functional difficulties among adults aged 15 years and older, with consistently higher rates among women (14). In Asia, Pakistan reports the highest prevalence at about 24%, with women (26.3%) more affected than men (21.6%). Afghanistan also shows a high burden, with 16.2% of adults experiencing functional difficulties, including 17.3% of women compared to 15.1% of men. In contrast, lower prevalence is observed in Bangladesh (8% overall; 8.9% women vs. 7% men) and Indonesia (5.3% overall; 5.8% women vs. 4.8% men) (14). Nigeria, included for broader LMIC comparison, reports a prevalence of 6.9%, with women (7.5%) slightly more affected than men (6.3%). In East Africa, a similar pattern is evident (14). Ethiopia reports a prevalence of 12.1%, with higher rates among women (12.9%) than men (11.3%). Rwanda shows 8.9% overall (9.6% women vs. 8.1% men), while Tanzania reports approximately 12%, with women (13.2%) more affected than men (10.8%) and prevalence increasing with age; vision and mobility limitations are among the most commonly reported (14). In Djibouti, 11.2% of adults report functional difficulties, with slightly higher prevalence among women (11.6%) than men (10.7%) (14). Overall, across LMICs in Asia and East Africa, functional difficulties vary widely in magnitude but consistently show higher prevalence among women, highlighting persistent gender disparities in disability burden (14).

Over a lifetime, many individuals experience temporary or long-term impairments, with the likelihood of functional limitations increasing with age. In many households, at least one person lives with a disability, often requiring care and support from family or community members. Societies and governments continue to face ongoing challenges in promoting inclusion and ensuring adequate support for persons with disabilities, particularly as populations age globally (15). Across the world, people with disabilities frequently encounter barriers that limit their participation in essential areas of life, including healthcare, education, and employment. Many also have limited access to supportive services and continue to face discrimination, marginalization, and social isolation. Following the adoption of the United Nations Convention on the Rights of Persons with Disabilities, disability has gained greater recognition as a fundamental human rights issue. At the same time, it is widely acknowledged as a major development challenge, as people with disabilities are more likely to experience economic hardship, reduced opportunities, and persistent poverty compared with individuals without disabilities (16).

In Somalia, persons with disabilities are often excluded from economic, social, and cultural life. Stigma, discrimination, limited accessibility, and systemic neglect contribute to poorer health, lower education levels, and reduced livelihood opportunities compared to those without disabilities (15). Families are also affected, as stigma and limited support services restrict participation and opportunity. Many children with disabilities are out of school and remain isolated, while both children and adults commonly experience social exclusion and stigma. These challenges are intensified by ongoing conflict, with girls and internally displaced persons with disabilities facing the greatest disadvantages (15). In response, the Somali government has introduced legal frameworks, policies, and social protection measures aimed at improving the living conditions of persons with disabilities (15). The Provisional Constitution of the Federal Republic of Somalia guarantees equal rights before the law and prohibits discrimination, including based on disability. Article 11 affirms that all citizens are entitled to equal rights and duties regardless of personal, social, or economic status, and recognizes discrimination as any action that undermines these rights (15, 17).

Conflict and weak healthcare systems have contributed to a relatively high burden of disability in Somalia. However, data on disability remain limited and incomplete, particularly among hard-to-reach populations such as nomadic communities. Although estimates suggest that up to 20% of the population may be living with a disability, exceeding global averages, the overall evidence base is weak. Existing studies are few and often focus on specific subgroups, such as children or individuals with psychosocial disabilities, with limited scope. As a result, there is a lack of comprehensive, nationally representative data on the prevalence and determinants of disability, particularly among women of reproductive age (15–49 years), leaving a significant gap in understanding disability distribution and its drivers in the country. To the best of our knowledge, evidence on disability among women aged 15–49 in Somalia remains limited, while most existing studies focus on specific aspects, particularly physical functioning, and are based on small, non-representative samples (18–20). Other research has addressed narrow themes such as unmet mental health needs among persons with disabilities (21), and access to disability-inclusive social protection services (22), while broader overviews remain limited (23). Overall, there is a clear lack of nationally representative data on the prevalence and determinants of disability among women of reproductive age in Somalia. This gap restricts understanding of the extent of disability and the barriers that affected women face, limiting the development of targeted interventions. Addressing this evidence gap is essential to inform policy, improve service delivery, and support monitoring and evaluation at national and geographical levels, including progress toward Sustainable Development Goal 3 on good health and well-being.

## Study approach and materials

### Context of the study

Somalia lies in the Horn of Africa and occupies an estimated 637,657 square kilometres. The country possesses the longest coastline on the African continent, stretching roughly 3,333 kilometres along the Gulf of Aden to the north and the Indian Ocean to the east and south. Somalia is bordered by Kenya in the southwest, Ethiopia in the west, and Djibouti in the northwest. Climatically, it experiences predominantly hot conditions with minimal seasonal variation, and average temperatures typically range from 30 °C to 40 °C (24). Administratively, Somalia is divided into eighteen regions, namely Awdal, Woqooyi Galbeed, Togdheer, Sool, Sanaag, Bari, Nugaal, Mudug, Galgaduud, Hiraan, Lower Shabelle, Middle Shabelle, Banadir, Bay, Bakool, Gedo, Lower Juba and Middle Juba. Somalia adopted a federal system of governance, comprising a Federal Government and several Federal Member States (17). The population of Somalia was estimated at 12,316,895 (25). In terms of residential distribution, 42.4% of the population resided in urban areas, 22.8% in rural areas, 25.9% led nomadic lifestyles, and 9.0% were internally displaced persons (IDPs). With respect to sex distribution, 50.7% of the population were male, and 49.3% were female (25). Agriculture and livestock production constitute the primary sources of livelihood for the Somali population (26). This study covered the entire territory of Somalia and incorporated urban, rural, and nomadic populations across all geographic areas.

### Data source and design

This study employed a quantitative cross-sectional study method using data from the 2020 Somalia Health and Demographic Survey (SHDS), which represents the first nationally representative household survey of its kind ever conducted in Somalia. The SHDS data collection was conducted between February 01, 2018, and January 31, 2019 (24). The Survey was designed as a nationally representative survey to produce valid, reliable, and comprehensive information on the demographic and health profile of the Somali population. Its primary objective was to generate robust evidence on population health and demographic characteristics to guide program development and the formulation of effective policies (24).

### Sampling procedures, sample size and weighting

The SHDS employed a stratified multistage cluster sampling design to produce representative estimates at the national, geographical, and residential levels (urban, rural, and nomadic populations) (24). With the exception of Banadir, which is entirely urban, each of the 18 pre-war administrative regions was stratified by place of residence, yielding 55 strata. Due to security limitations, several strata were excluded, reducing the final sampling frame to 47 strata. Enumeration Areas (EAs) served as the primary sampling units, while Temporary Nomadic Settlements (TNSs) were used for nomadic populations. In urban and rural areas, a three-stage stratified cluster sampling approach with probability proportional to size (PPS) was applied, whereas a two-stage stratified cluster design was used in nomadic areas (24). Overall, 1,433 EAs were selected from the sampling frame, and households within selected clusters were systematically sampled to ensure adequate spatial representation. A total of 538 Enumeration Areas (EAs) were included in the survey, resulting in a sample of 16,360 households. Of the selected households, 15,826 were successfully interviewed, yielding a response rate of 99.7%. Sampling weights were applied to account for selection probability and non-response adjustments (24). Accordingly, the primary units of analysis for the survey were households, women aged 15–49 years, and children aged 0–5 years (24). For our study, the target population included all women of reproductive age (15–49 years), and the final analytical sample comprised 18,155 women within this age group.

## Study outcome and determinants

### Dependent variable

Disability status served as the primary exposure variable in this study and was ascertained through questions adapted from the Washington Group Short Set on Functioning (WG-SS), an internationally validated measurement tool that was incorporated as a standard component of the 2018/2019 SHDS (27). Respondents were systematically assessed across seven core functional domains, encompassing seeing, hearing, mobility, learning, self-care, speech and mental (27). Responses within each domain were captured on a four-point ordinal scale, ranging from no difficulty, some difficulty, and a lot of difficulty, to cannot do it at all. In accordance with the Washington Group’s recommended classification protocol, women who reported experiencing “a lot of difficulty” or “cannot do it at all” in at least one of the seven functional domains were categorized as having a disability, whereas those who indicated “no difficulty” or “some difficulty” across all domains were classified as having no disability (27). Accordingly, disability status was operationalized as a binary outcome variable in the analysis, coded as N = 0 for women without any form of disability and Yes = 1 for women with at least one functional limitation, consistent with the study’s first objective of determining the prevalence of disability among women of reproductive age (15–49 years). Respondents were permitted to report more than one type of disability; therefore, the categories are not mutually exclusive. In addition, the binary outcome variable (any disability) was defined as the presence of at least one reported disability type among the seven categories assessed in the survey. This classification was based on existing literature on the seven types of disability, generated from the survey data regarding complete information on the items (28–30).

### Independent variable

Explanatory variables were organized into two categories: individual-level and community-level factors guided by evidence from prior studies. All selected variables were drawn from the 2018/2019 Somalia Health and Demographic Survey (SHDS 2018/2019) dataset (24) (Table 1).

**Table 1:** Independent variables and descriptions.

Level	Variable	Options
Individual-level factors		
	Age in years	1 = 15–24 years, 2 = 25–34 years, 3 = 35–49 years (45).
	Marital status	1 = Married, 2 = Divorced, 3 = Abandoned, 4 = Widowed, 5 = Never Married (29)
	Woman’s education	1 = Uneducated 2 = Educated (46); (47).
	Household size	1–5 members, 6–10 members, ≥11 members (46).
	Media exposure	1 = Yes, 2 = No (J. (48)).
	Health coverage	1 = Yes, 2 = No (46).
Community-level factors		
	Wealth quintile	1 = Lowest, 2 = Second, 3 = Middle, 4 = Fourth, 5 = Highest (46)
	Residence	1 = Urban 2 = Rural (49).
	Geographical areas	1 = North 2 = Central 3 = South (50).

### Statistical data analysis

The unit of analysis comprised women of reproductive age, specifically those aged 15 to 49 years. All data extraction, recoding, and subsequent statistical analyses were performed using Stata version 17.0. To ensure methodological rigor, the complex survey design of the 2018/2019 SHDS was explicitly accounted for prior to analysis through the specification of the sampling structure via the svyset command. Sampling weights (wgt) were applied to correct for unequal probabilities of selection, thereby ensuring that the estimates derived from the analysis were nationally representative of the target population. The multi-stage nature of the survey design was further addressed by designating the Enumeration Area (EA) as the primary sampling unit (PSU), which appropriately captured the clustered structure of the data. Variance estimation was subsequently carried out using a design-consistent variance–covariance estimation (VCE) approach, which ensured that the resulting standard errors faithfully reflected the true complexity of the sampling framework. This approach was essential to avoid the inferential biases that would otherwise arise from the erroneous assumption of simple random sampling. Descriptive statistics were first calculated to summarize the study population’s characteristics using frequencies and percentages. Bivariate logistic regression was then performed to examine associations between each predictor variable and the prevalence of disability among women aged 15–49 years. Variables with a *p*-value ≤ 0.25 in the bivariate analysis were selected for inclusion in the multilevel logistic regression analysis. A multilevel multivariable logistic regression approach was used to identify predictors of disability status among women aged 15–49 years. This analysis followed four models: The empty model (involved outcome variable only), Model I included only individual-level variables; Model II included only community-level variables; and Model III included both individual- and community-level variables simultaneously. The associations between individual-level variables and disability were assessed in Model I, while Model II assessed community-level variables. Model III combined both levels of variables to evaluate their joint effects on disability status. Finally, variables with *p*-values less than 0.05 and adjusted odds ratios (AOR) with 95% confidence intervals (CIs) were considered statistically significant predictors of disability status. Multicollinearity among the independent variables was assessed using the Variance Inflation Factor (VIF). All VIF values were below the conventional threshold of 10, with a mean VIF of 1.43, indicating no evidence of multicollinearity among the predictor variables included in the model. This confirms that the regression estimates are reliable and free from multicollinearity concerns. A sensitivity analysis was performed using Firth penalized likelihood logistic regression to assess the robustness of the estimated association between health insurance coverage and disability. This approach was applied to address potential small-sample bias and rare-event issues due to the low number of insured women with disability. The Firth method reduces estimation bias in maximum likelihood models, particularly in cases of sparse data or quasi-separation.

### Random effects analysis

To quantify the extent of clustering and between-cluster variation in disability among women of reproductive age, three complementary measures of random effects were computed: The Intraclass Correlation Coefficient (ICC), the Proportionate Change in Variance (PCV), and the Median Odds Ratio (MOR). The ICC was calculated to assess the proportion of the total variance in disability attributable to between-cluster differences, and was derived using the following formula.


$$\mathrm{ICC}=\frac{\delta^{2}}{\delta^{2}u^{0}+\pi^{2}/3}\times 100\%$$

Where $\delta^{2}u^{0}$ represents the cluster-level variance and $\pi^{2}/3\;$ (approximately 3.29) is the standard variance of the logistic distribution.

The MOR was computed to quantify the unexplained heterogeneity between clusters by estimating the median value of the odds ratio between the highest-risk and lowest-risk clusters for disability. The MOR was calculated as follows.


$$\mathrm{MOR}=\exp(0.6745\sqrt{\delta^{2}u^{0}})$$

A MOR value greater than 1 indicates the presence of contextual-level variation, with values further from 1 reflecting greater between-cluster heterogeneity.

The PCV was additionally calculated to quantify the proportion of the total cluster-level variance explained by the inclusion of covariates across successive models, using the following formula.


$$\mathrm{PCV}=\frac{\delta^{2}u^{0}-\delta^{2}}{\delta^{2}u^{0}}\times 100\%$$

Where $\delta^{2}u^{0}$ denotes the variance of the null model (Model 0) and $\delta^{2}$ represents the residual cluster-level variance in each successive model. A higher PCV value indicates that a greater proportion of the between-cluster variation in disability is explained by the covariates included in the respective model.

### Model development and selection of the best-fitted model

**Figure 1:**
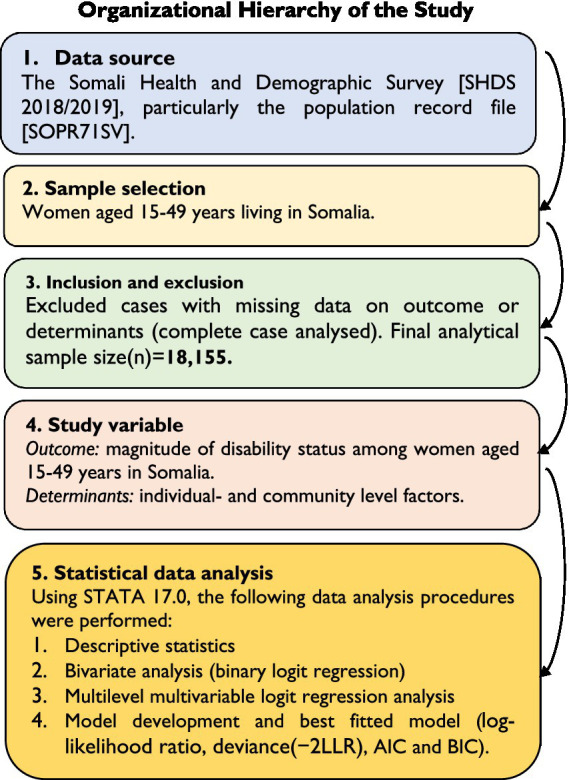
Study flowchart developed by the authors.

Model comparison and goodness of fit were evaluated using the Akaike Information Criterion (AIC), Bayesian Information Criterion (BIC), and log-likelihood (LLR) values. The model with the lowest AIC and BIC was considered to provide the best balance between model fit and complexity. Although AIC is commonly preferred for selecting models with strong predictive performance and applies a relatively less stringent penalty for complexity in large samples (*n* = 18,155), BIC favours more parsimonious models by imposing a stronger penalty for additional parameters (Figure 1).

## Results of the study

### Prevalence of disability among women aged 15–49 years in Somalia

Table 2 presents the prevalence of disability among women of reproductive age (15–49 years) in Somalia. A total of 18,155 women were included in the analysis; the overwhelming majority (96.95%) reported no form of disability, while a comparatively smaller proportion (3.05%) was identified as living with at least one form of disability.

**Table 2:** Prevalence of disability among women aged 15–49 years in Somalia [*n* = 18,155].

Disability status	*N*	Prevalence	Std. err.	[95% conf. interval]
No, disability	17,602	96.95%	0.0013	[0.967, 0.972]
Yes, any disability	553	3.05%	0.0013	[0.028, 0.033]

### Demographic, socioeconomic, and geographic profile of women aged 15–49 years in Somalia

Table 3 reports the demographic, socioeconomic, and geographic characteristics of women aged 15–49 years in Somalia. The average age of the women was 26 with SD (±8 years). Most respondents were married 62.02%, followed by those who had never married 29.93%, while smaller proportions were divorced 5.79%, widowed 1.90%, or abandoned 0.36%. In terms of educational attainment, nearly three-quarters of the women were uneducated 74.02%, whereas 25.98% had attained some level of education. Regarding household composition, more than half of the respondents lived in households with 6–10 members 54.08%, followed by households with 1–5 members 33.71, and 12.21% resided in households with ≥11 members. Exposure to mass media was limited, with 69.99% of women reporting no access, while 30.01% had some exposure. Health insurance coverage was extremely low; only 0.46% of respondents reported having any form of health insurance, compared with 99.54% who had none. Socioeconomic status, as measured by wealth quintiles, showed that 31.30% of women belonged to the lowest wealth quintile, while the remaining were distributed across the second 15.87%, middle 17.80%, fourth 17.85%, and highest 17.18% quintiles. In residence, slightly more than half of the respondents resided in rural areas 51.74%, while 48.26% lived in urban settings. Geographically, the majority were from the Northern areas, 50.54%, followed by the Southern 28.56% and the Central 20.90%.

**Table 3:** Demographic, socioeconomic, and geographic profile of women aged 15–49 years.

Individual-level variable	Frequency	Percent
Age in years		
15–24 years	8,436	46.47
25–34 years	5,624	30.98
35–49 years	4,095	22.56
Marital status		
Married	11,259	62.02
Divorced	1,052	5.79
Abandoned	65	0.36
Widowed	345	1.90
Never married	5,434	29.93
Women’s education		
Uneducated	13,439	74.02
Educated	4,716	25.98
Household size		
1–5 members	6,120	33.71
6–10 members	9,819	54.08
≥11 members	2,216	12.21
Media exposure		
No	12,707	69.99
Yes	5,448	30.01
Health insurance coverage^a^		
Yes	83	0.46
No	18,070	99.54
Wealth quintile		
Lowest	5,683	31.30
Second	2,882	15.87
Middle	3,231	17.80
Fourth	3,240	17.85
Highest	3,119	17.18

Community level	Frequency	Percent
Place of residence		
Urban	8,761	48.26
Rural	9,394	51.74
Geographical areas		
North	9,175	50.54
Central	3,795	20.90
South	5,185	28.56

a The sample size for the health insurance coverage variable was (*n* = 18,153) because of missing observations. We also operationalized the regions by grouping them into three geographical areas: North (Awdal, Woqooyi Galbeed, Togdheer, Sool, Sanaag, Bari, and Nugaal), Central (Mudug, Galgaduud, Hiraan, and Middle Shabelle), and South (Banadir, Bay, Bakool, Gedo, and Lower Juba) due to the large number of options and small size to facilitate analysis and improve interpretability (51).

### Distribution of disability types among women aged 15–49 years in Somalia

**Figure 2:**
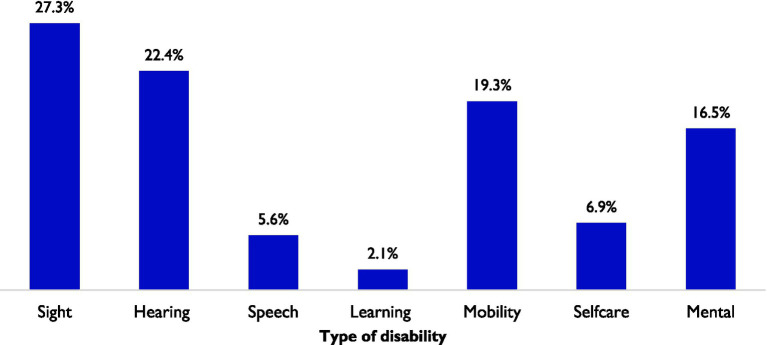
Distribution of disability types among women aged 15–49 years in Somalia.

Figure 2 presents the distribution of disability types among women aged 15–49 years in Somalia across seven categories: sight, hearing, speech, learning, mobility, self-care, and mental disabilities. Sight-related disabilities were the most commonly reported (27.3%), followed by hearing impairments (22.4%). Mobility limitations accounted for 19.3%, while mental disabilities were reported by 16.5% of women. Self-care difficulties were reported by 6.9%, speech-related disabilities by 5.6%, and learning disabilities were the least prevalent at 2.1%.

## Bivariate logit regression analysis

### Bivariate analysis of disability and its determinants among women aged 15–49 years in Somalia

Table 4 presents the bivariate association between disability status and selected sociodemographic characteristics among women aged 15 to 49 years in Somalia. Disability was significantly associated with all the examined determinants, with a *p*-value less than 0.25. Women aged 35–49 years had the highest prevalence of disability at 4.0%, while the youngest group aged 15–24 years had the lowest at 2.79%. Disability status was most common among abandoned women at 9.26% and widowed women at 6.71%, whereas married women had the lowest prevalence at 2.02%. Women with no formal education reported a higher prevalence of disability at 3.25% compared to 2.46% among those with education. Prevalence was slightly higher in smaller households with 1–5 members at 3.10% and in the largest households with ≥11 members at 2.98%, compared to mid-sized households. Slightly higher disability prevalence was observed among women with media exposure at 3.19% than among those without at 2.98%. Women with health insurance reported a higher prevalence at 4.82% compared to uninsured women at 3.04%. Women in the fourth and middle wealth quintiles had slightly higher disability at 3.80 and 3.53%, respectively, compared to women in the lowest quintile at 2.23% and the highest quintile at 3.01%. Urban women had a higher disability prevalence at 3.69% than rural women at 2.45%. Finally, prevalence was similar in the northern and southern areas at 3.12% and slightly lower in the central location at 2.77%.

**Table 4:** Bivariate analysis of disability and associated determinants among women aged 15–49 years.

Variable	Category	Disability status				*ρ*-value
		Yes		No		
		%	[95% CI]	%	[95% CI]	
Age in years						<0.001
	15–24 years	2.79	[0.025, 0.032]	97.21	[0.968, 0.975]	
	25–34 years	2.74	[0.023, 0.032]	97.26	[0.968, 0.977]	
	35–49 years	4.00	[0.034, 0.047]	96.00	[0.953, 0.966]	
Marital status						<0.001
	Married	2.02	[0.018, 0.023]	97.98	[0.977, 0.982]	
	Divorced	5.01	[0.037, 0.067]	94.99	[0.933, 0.963]	
	Abandoned	9.26	[0.039, 0.204]	90.74	[0.796, 0.961]	
	Widowed	6.71	[0.044, 0.102]	93.29	[0.898, 0.956]	
	Never Married	3.92	[0.034, 0.045]	96.08	[0.955, 0.966]	
Women’s education						<0.001
	Uneducated	3.25	[0.030, 0.036]	96.75	[0.964, 0.970]	
	Educated	2.46	[0.021, 0.029]	97.54	[0.971, 0.979]	
Household size						<0.001
	1–5 members	3.10	[0.027, 0.036]	96.90	[0.964, 0.973]	
	6–10 members	3.02	[0.027, 0.034]	96.98	[0.966, 0.973]	
	≥11 members	2.98	[0.023, 0.038]	97.02	[0.962, 0.977]	
Mass media						<0.001
	No	2.98	[0.027, 0.033]	97.02	[0.967, 0.973]	
	Yes	3.19	[0.028, 0.037]	96.81	[0.963, 0.973]	
Health insurance						<0.001
	Yes	4.82	[0.018, 0.121]	95.18	[0.879, 0.982]	
	No	3.04	[0.028, 0.033]	96.96	[0.967, 0.972]	
Wealth index						<0.001
	Lowest	2.23	[0.019, 0.027]	97.77	[0.973, 0.981]	
	Second	3.30	[0.027, 0.040]	96.70	[0.960, 0.973]	
	Middle	3.53	[0.029, 0.042]	96.47	[0.958, 0.971]	
	Fourth	3.80	[0.032, 0.045]	96.20	[0.955, 0.968]	
	Highest	3.01	[0.025, 0.037]	96.99	[0.963, 0.975]	
Residence						<0.001
	Urban	3.69	[0.033, 0.041]	96.31	[0.959, 0.967]	
	Rural	2.45	[0.022, 0.028]	97.55	[0.972, 0.975]	
Geographical areas						<0.001
	North	3.12	[0.028, 0.035]	96.88	[0.965, 0.972]	
	Central	2.77	[0.023, 0.033]	97.23	[0.967, 0.977]	
	South	3.12	[0.027, 0.036]	96.88	[0.964, 0.973]	

### Prevalence of disability by type of residence among women aged 15–49 years in Somalia

**Figure 3:**
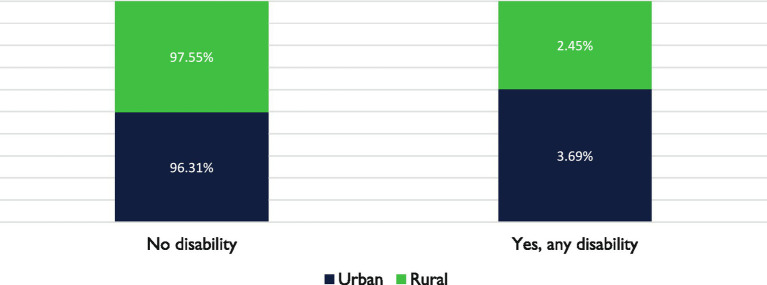
Prevalence of disability by type of residence among women aged 15–49 years in Somalia (%).

Figure 3 proves the distribution of disability status by place of residence among women aged 15–49 years in Somalia. The disaggregation by residence shows slight differences between urban and rural areas. In urban settings, 96.31% of women reported no disability, compared to 3.69% who had some form of disability. In rural areas, the proportion of women without disabilities was slightly higher at 97.55%, while 2.45% reported having a disability. These findings indicate a marginally higher prevalence of disability among women in urban areas compared to their rural counterparts.

## Multilevel multivariable logistic regression analysis

### Multivariable multilevel logit regression model across individual- and community-level determinants of disability

Table 5 presents the results of the multivariable multilevel logistic regression analysis assessing individual- and community-level determinants of disability. Only statistically significant variables are interpreted. At the individual level, age was a significant predictor of disability. Compared with individuals aged 15–24 years, those aged 25–34 years had significantly higher odds of disability (AOR = 1.42, 95% CI: 1.11–1.81), while the odds were even higher among those aged 35–49 years (AOR = 2.17, 95% CI: 1.67–2.83), indicating an increasing likelihood of disability with advancing age. Marital status also showed a strong and consistent association with disability. Compared with married individuals, those who were divorced (AOR = 2.34, 95% CI: 1.73–3.17), abandoned (AOR = 4.99, 95% CI: 2.33–10.69), widowed (AOR = 2.20, 95% CI: 1.39–3.50), and never married (AOR = 3.36, 95% CI: 2.63–4.30) had significantly higher odds of disability, suggesting increased vulnerability among individuals outside marital unions. Educational attainment was inversely associated with disability. Educated women were significantly less likely to have a disability compared with uneducated women (AOR = 0.51, 95% CI: 0.40–0.64), highlighting the protective role of education. Household size was another significant determinant. Individuals living in households with 6–10 members (AOR = 0.71, 95% CI: 0.58–0.84) and those in households with 11 or more members (AOR = 0.67, 95% CI: 0.47–0.85) had significantly lower odds of disability compared with those living in smaller households (1–5 members). Women without health insurance had significantly lower odds of reported disability compared with insured women in the main model (AOR = 0.04, 95% CI: 0.03–0.05). This lower reported odds of disability among uninsured women likely reflect detection and reporting bias, as insured women have greater access to healthcare services, clinical assessment, and formal diagnosis, while uninsured women face financial and structural barriers that limit healthcare utilization, resulting in underdiagnosis and underreporting of disability. However, this association was not confirmed in sensitivity analysis using Firth penalized likelihood logistic regression, which showed a non-significant effect (OR = 0.554, 95% CI: 0.214–1.438; *p* = 0.225). With respect to household economic status, individuals in the highest wealth quintile were significantly less likely to have a disability compared with those in the lowest quintile (AOR = 0.63, 95% CI: 0.44–0.90), indicating a protective effect of higher socioeconomic status. Finally, the community level, place of residence, was significantly associated with disability. Rural residents had lower odds of disability compared with urban residents (AOR = 0.45, 95% CI: 0.36–0.56). Geographical areas were also an important determinant; individuals residing in the southern areas had significantly lower odds of disability compared with those in the northern (AOR = 0.75, 95% CI: 0.61–0.92).

**Table 5:** Multilevel multivariable logistic regression model of individual- and community-level determinants.

Variable	Model 0 [only outcome variable]	Model I [individual level factors]	Model II [community level factors]	Model III [individual and community level factors]
		AOR [95% CI]	AOR [95% CI]	AOR [95% CI]
Age in years				
15–24 years		1		1
25–34 years		1.30 (1.02–1.66)*		1.42 (1.11–1.81)*
35–49 years		1.95 (1.50–2.53)*		2.17 (1.67–2.83)*
Marital status				
Married		1		1
Divorced		2.31 (1.70–3.13)*		2.34 (1.73–3.17)*
Abandoned		5.27 (2.47–11.23)*		4.99 (2.3310.69)*
Widowed		2.17 (1.37–3.46)*		2.20 (1.39–3.50)*
Never Married		3.13 (2.46–3.99)*		3.36 (2.63–4.30)*
Woman’s education				
Uneducated		1		1
Educated		0.50 (0.40–0.64)*		0.51 (0.40–0.64)*
Household size				
1–5 members		1		1
6–10 members		0.71 (0.59–0.85)*		0.70 (0.58–0.84)*
≥11 members		0.67 (0.50–0.90)*		0.63 (0.47–0.85)*
Media exposure				
No		1		1
Yes		0.95 (0.76–1.19)		0.92 (0.73–1.15)
Health insurance				
Yes		1		1
No		0.02 (0.02–0.02)*		0.04 (0.03–0.05)*
Wealth quintile				
Lowest		1		1
Second		1.13 (0.87–1.47)		0.88 (0.67–1.17)
Middle		1.28 (1.00–1.64)*		0.88 (0.67–1.17)
Fourth		1.37 (1.06–1.78)*		0.85 (0.63–1.14)
Highest		1.02 (0.73–1.42)		0.63 (0.44–0.90)*
Residence				
Urban			1	1
Rural			0.04 (0.04–0.05)*	0.45 (0.36–0.56)*
Geographical areas				
North			1	1
Central			0.07 (0.06–0.09)*	0.81 (0.64–1.02)
South			0.05 (0.04–0.05)*	0.75 (0.61–0.92)*

The asterisk (*) indicates statistical significance at *ρ* < 0.05 in the multilevel analysis

### Model comparison, goodness-of-fit, and random-effects analysis of determinants associated with disability among women of reproductive age

Table 6 presents the random-effects analysis and model fitness comparison. To determine the appropriateness of multilevel modeling, a null (empty) model was initially fitted. The null model demonstrated significant between-cluster variation in disability, with a random intercept variance of 0.1043 and a statistically significant likelihood ratio test (*p* < 0.001), confirming the presence of meaningful clustering across communities and justifying the use of a multilevel analytical approach.

**Table 6:** Model comparison, goodness-of-fit, and random-effects analysis of determinants associated with disability among women of reproductive age.

Parameter	Model 0	Model I	Model II	Model III
Variance	0.1043	0.1568	0.1102	0.0755
ICC	3.10%	4.60%	3.20%	2.20%
MOR	1.36	1.46	1.37	1.30
PCV	Reference	−50.40%	−5.70%	27.50%
Model fitness				
LLR	−2460.79	−2434.16	−4522.121	−2404.58
Deviance (−2LLR)	4921.58	4868.32	9044.24	**4809.15**
AIC	4923.58	4898.32	9050.24	**4845.16**
BIC	4931.39	5015.44	9073.66	**4985.70**

ICC, intra-cluster correlation; PCV, proportional change in variance; MOR, median odds ratio; LLR, log-likelihood ratio; AIC, Akaike Information Criterion; BIC, Bayesian Information Criterion; The asterisk (*) indicates statistical significance at *ρ* < 0.05 in the multilevel analysis. Bold values denote the best-fitted model based on the lowest deviance, AIC, and BIC values.

The intraclass correlation coefficient (ICC) further indicated that part of the total variation in disability was attributable to differences between clusters, with ICC values of 3.1% in the empty model, 4.6% in Model I, 3.2% in Model II, and 2.2% in the fully adjusted Model III. The reduction in ICC values across successive models suggests that the inclusion of both individual- and community-level covariates progressively explained the observed between-cluster heterogeneity in disability, particularly in the final model. Likewise, the proportional change in variance (PCV) showed that Model III explained the largest proportion of cluster-level variation (27.5%) relative to the null model, indicating improved explanatory capacity after accounting for both individual and contextual determinants. In contrast, the negative PCV values observed in Model I (−50.4%) and Model II (−5.7%) suggest that these intermediate models did not adequately explain the cluster-level variance and may have introduced additional unexplained heterogeneity. Overall, these findings indicate that substantial community-level differences in disability existed and that the fully adjusted model reduced much of the unexplained variation across clusters.

Model comparison and fitness were assessed using deviance statistics, calculated as −2 times the log-likelihood ratio (−2LLR), whereby models with lower deviance values were considered to provide better fit. In addition, the Akaike Information Criterion (AIC) and Bayesian Information Criterion (BIC) were employed to evaluate model fit while accounting for model complexity and parsimony. The fully adjusted model incorporating both individual- and community-level covariates (Model III) demonstrated the lowest deviance (4809.15), AIC (4845.16), and BIC values among all competing models, indicating superior explanatory performance and the most parsimonious fit to the data. These findings suggest that simultaneously accounting for both individual and contextual determinants provided the most comprehensive explanation of disability among women of reproductive age in Somalia.

## Discussion

This study explored the prevalence and determinants of disability among women of reproductive age and identified several significant associated factors. The findings revealed that 3.05% of women aged 15–49 years reported at least one form of disability. This prevalence is comparable to the 4.2% national disability prevalence reported in Guatemala (31) and lower than the 14.1% observed in Pakistan (32). In addition, evidence from 14 low- and middle-income countries indicates substantial variability in mobility-related disability among women of reproductive age, ranging from 0.32 to 21.45% (11). The observed variation in disability prevalence across studies may be attributed to differences in socioeconomic conditions, burden of chronic disease, exposure to conflict and injuries, nutritional and maternal health status, healthcare access, cultural perceptions and reporting of disability, as well as methodological differences in sampling procedures, disability definitions, measurement approaches, and study populations.

Multilevel logistic regression analysis showed that older women were more likely to report disability. Women aged 25–34 years had 1.4 times higher odds of disability, while those aged 35–49 years were more than twice as likely to experience disability compared with women aged 15–24 years. This finding is consistent with previous studies (11, 29), and may be explained by the cumulative impact of age-related health conditions, repeated reproductive burden, chronic disease exposure, and prolonged limitations in access to preventive and curative healthcare services over the life course. In contrast, marital status was significantly associated with disability among women of reproductive age in Somalia. Compared with married women, abandoned women were 4.99 times more likely to report disability, followed by never-married women (3.36 times), divorced women (2.34 times), and widowed women (2.20 times). These findings suggest that the absence of spousal support may increase vulnerability to disability through greater exposure to economic hardship, psychosocial stress, social isolation, and limited access to healthcare and caregiving support. In contrast, marriage may provide protective benefits through enhanced social, emotional, and financial stability. These results are consistent with previous studies linking marital disruption and lack of spousal support to increased disability risk among women (30, 33). Regarding educational status, women with no formal education were 0.51 times less likely to report disability compared with educated women. This finding may reflect differences in health awareness, recognition of functional limitations, and reporting behaviour, as educated women may be more likely to identify and report disability-related conditions. This remark is consistent with findings from previous studies (28, 34, 35).

On the contrary, women with health insurance status were 0.04 times less likely to report disability compared with uninsured women. This finding may reflect underdiagnosis and limited healthcare utilization among uninsured women, as women with health insurance are more likely to access healthcare services, receive medical evaluations, and report disability-related conditions. This study is corroborated by findings from previous studies (7, 36, 37). Similarly, household size, women living in medium-sized households (6–10 members) and large households (≥11 members) were 0.71 and 0.67 times less likely to report disability, respectively, compared with those living in smaller households. This finding may reflect the protective role of larger family networks through shared household responsibilities, increased social support, and greater caregiving capacity, which may reduce vulnerability to disability. This finding is in line with findings from previous studies (29, 38). Alternatively, economic status was associated with disability, where women in the highest wealth quintile were 0.63 times less likely to report disability compared with those in the lowest quintile. This finding may reflect the protective role of higher socioeconomic status through improved access to healthcare services, better nutritional status, and safer living and working conditions, which may collectively reduce vulnerability to disability. This observation is consistent with findings from previous studies (13, 39–41).

Furthermore, differences by place of residence were observed, with women in rural areas showing lower odds of reported disability (AOR = 0.45) compared with their urban counterparts. This pattern may be explained by contextual and measurement-related factors, including limited access to healthcare services in rural settings, which may contribute to underdiagnosis and underreporting of functional limitations. In addition, differences in occupational exposure, more physically demanding rural livelihoods, and lower levels of health literacy may reduce awareness and reporting of disability, whereas greater access to diagnostic and health services in urban areas may increase detection and reporting. These findings are consistent with evidence from previous studies (42–44). Finally, geographical disparities were observed, where women residing in the South were 0.75 times less likely to report disability compared with those in the North areas. This finding may reflect geographical differences in health infrastructure, exposure to conflict, and availability of social services. Moreover, persistent insecurity in the South may further limit access to healthcare services, thereby reducing diagnosis and reporting of functional limitations. Which may reflect geographical disparities in health infrastructure, exposure to conflict, availability of social services, and the persistent insecurity in the South that limits access to healthcare.

## Limitations

This study provides important insights into disability among women of reproductive age, but several limitations should be noted. Reliance on self-reported data may lead to underestimation due to social desirability bias, and the cross-sectional design limits the ability to establish causal relationships with factors such as age, marital status, education, wealth, media exposure, health insurance, residence, and geographic areas. Geographic masking of survey clusters, particularly in nomadic areas, and the exclusion of conflict-affected populations may have introduced spatial misclassification and selection bias. Additionally, we acknowledge that the binary classification of disability does not fully capture its complexity, as it masks variation in severity and the number of functional domains affected, and may therefore underrepresent differences in health status and chronic conditions, while unmeasured contextual factors such as clan dynamics and informal support networks may also have influenced the observed associations.

## Conclusions and policy implications

This study underscores that disability among women of reproductive age in Somalia is shaped by both individual and community-level factors, highlighting the need for integrated, multi-sectoral interventions to improve education, healthcare access, and social protection for vulnerable women. Based on the findings, the following key policy priorities are recommended:

- *Strengthen equitable access to healthcare services:* Expand community-based and publicly funded primary healthcare and scale up mobile and outreach services, as low insurance coverage and reliance on out-of-pocket payments limit access and likely contribute to underdiagnosis of disability.
- *Improve disability detection and reporting systems:* Strengthen routine health information systems and reduce reliance on facility-based diagnosis by integrating community-level screening to address underreporting, particularly among uninsured populations.
- *Target high-risk demographic and social groups:* Prioritize age-sensitive screening and rehabilitation for women aged 25–49 years and provide targeted support for women experiencing marital disruption, who show higher vulnerability to disability.
- *Enhance education and socioeconomic empowerment:* Invest in female education and health literacy, and promote economic empowerment and poverty reduction strategies, as higher education and wealth are associated with lower odds of disability.
- *Reduce geographic inequalities:* Address significant urban–rural and geographical disparities through improved rural health infrastructure, outreach services, and strengthened community health worker programs.

## Data Availability

The datasets presented in this study can be found in online repositories. The names of the repository/repositories and accession number(s) can be found at: https://microdata.nbs.gov.so/index.php/catalog/59/get-microdata.
